# Digital Image Recognition Based on Improved Cognitive Neural Network

**DOI:** 10.1515/tnsci-2019-0021

**Published:** 2019-04-23

**Authors:** Yuxi Liu

**Affiliations:** 1 Computing and IT, College of Sciences and Engineering, University of Tasmania in Australia, Hobart, Tasmania 7000, Australia

**Keywords:** Cognitive neural network, corresponding digital matrix, digital image recognition

## Abstract

This paper presents an innovative cognitive neural network method application in digital image recognition. The following conclusion can be drawn. Each point of the graph is transformed, and the original color of the transformed new coordinates is given to the point. If after all the points have transformed, if there is a point and no point has converted to this point, the point is not given a color. Then this point will form a hole or a stripe, and the color is the color of the point initialization. The innovative method can effectively separate the digital image recognition signal from the mixed signal and maintain the waveform of the source signal with high accuracy, thus laying the foundation for the next step of recognition.

## 1 Introduction

In recent years, many scholars have conducted many discussions on the topic of digital image recognition and have proposed many methods. Aiming at the confusing characteristics of the feature vectors of handwritten numerals in the literature, a handwritten digital recognition method based on a combination of quantum methods, and multi-class classifiers based on multi-layer excitation functions has proposed. Use MNIST training and testing. This recognition method has a good effect in terms of recognition rate and reliability [1]. In the literature, the clustering technology and genetic algorithm have combined, and a method integration method based on similarity propagation algorithm and genetic algorithm has applied to handwritten digit recognition. In the literature, the characteristics of wavelet transform a in image processing has proposed a new offline handwritten digital feature extraction of local Fourier transform.

In this paper, digital image recognition system has designed based on RBF method. An innovative algorithm is an adaptive learning rate of adjustment algorithm, so that the learning rate of the network weight and threshold adjustment process can be based on the network error surface. The curvature of the different regions changes adaptively. In this paper, the digital pictures used for training and testing have read and binarized to form a corresponding digital matrix [2]. Then the matrix used for training has brought into the method for weights and threshold. The test result of the innovative RBF method has a higher recognition rate in digital image recognition.

## 2 Grayscale image and binarization

### 2.1 Grayscale image

In the computer field, a grayscale digital image is an image with only one sample color per pixel. Unlike grayscale images and black and white images, black and white images in computer graphics have only black and white colors; grayscale images have many levels of color depth between black and white [3]. The grayscale image used for display has usually saved with a non-linear scale of 8 bits per sampled pixel, which can have 256 levels of grayscale.

### 2.2 Binarization

For grayscale images, choose one or more grayscale values t (0[t[255]) to separate the target from the background [4]. This grayscale value has called the threshold. If only one threshold has selected, it has called image binarization. Binarization has also called gray scale division. Binarization is to set the gray value of the pixel on the image to 0 or 255, that is, to present the entire image with a distinct black and white visual effect.

## 3 Cognitive neural network

The learning process of the cognitive neural network method is different from the BP way. The hidden layer in the cognitive neural network is a sigmoid function whose value is a non-zero value in an infinitely large range in the input space [4]. The cognitive neural network method is a global approximation network with three-layer forward network.

According to the empirical formula, the step-by-step test method determines the number of hidden layer nodes, and gradually calculates the input vector and the corresponding center distance, and then gradually increases the value based on this value. By comparing the entire RBF network can predict the network performance, choose the best performance [5, 6]. The number of nodes corresponding to the entire RBF network can be used as the number of hidden layer neuron nodes.

(1) $$f\left(\text{z}\right)=\exp\;o\left(-\frac{{\left\|z-c\right\|}^{2}}{2\delta^{2}}\right)$$

**Figure 1:**
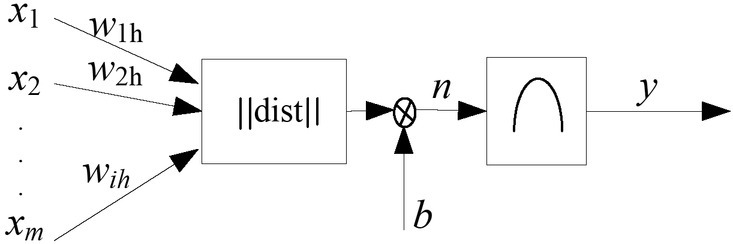
The structure of cognitive neural network method

Figure 1 shows the neuronal work flow of cognitive neural network.

The cognitive neural network architecture is based on two points: 1. Information is stored on the network through the distribution of excited patterns on the neurons; 2. Information processing is accomplished through dynamic processes of simultaneous interaction between neurons. The radial basis network mathematical model is the network input IV training samples.

(2) $$z_{\text{k}}=\left\{z_{\text{k1}},z_{\text{k2}}\ldots .z_{\text{km}}\right\}\;\;\;\;\;\;\;\;\;\;\;\;\;\;\;\;\;\left(k=1,2,\ldots N\right)$$

Network output:

(3) $$Y_{\text{k}}=\left\{y_{\text{k1}},y_{\text{k2}}\ldots .y_{\text{km}}\right\}\;\;\;\;\;\;\;\;\;\;\;\;\;\;\;\;\;\;\;\;\;\;\;\;\left(k=1,2,\ldots N\right)$$

The network output connection weights form a connection matrix.

(4) V={m,n},   m=1,2,….M,   n=1,2,….N

Hidden node number I, center point: {*C_i_*}*i =* 1,2,... *I* variance is

The relationship between network input and output:

(5) $$y_{kj}\left(z_{k}\right)=\sum_{m=1}^{M}V_{mn}\exp{\left(-\frac{1}{2\delta m^{2}}\left\|X_{k}-C_{n}\right\|\right)}^{2}$$

The performance index function of the cognitive neural network approximation is as follows:

(6) $$E\left(k\right)=\frac{1}{2}{\left[Y\text{m}-y_{mn}\left(X_{m}\right)\right]}^{2}$$

### 3.1 The reason for data preprocessing

The numbers in the input image cannot be directly used as test inputs. Digital strokes in images are sometimes irregular and may overlap. Because the examples in this article are screenshots to simplify the use, location distortion correction, color brightness correction, etc. have omitted, but still need some simple processing. The following is a simple pre-processing of the input image. The main purpose is to separate the two numbers. The method is very simple. First, the image has converted into a binary image, and then etched, and the numbers have separated from each other. This facilitates our next division and identification. This has the added benefit of removing the rest of the noise [7]. Therefore, we need to initialize the data sample, map all the sample data to certain limited space according to certain rules, and then use it to train the network, which can achieve good results.

### 3.2 Data preprocessing method

**Figure 2:**
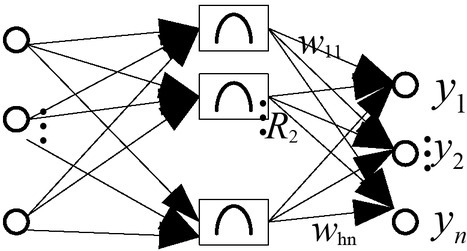
The radial basis method structure

This article segmented the image. Since our classifiers can only recognize numbers one by one, we must first separate each number. The basic idea is to first find out the basic contour with the findContours() function and then verify it with simple verification to see if it is a digital contour. For those validated outlines, boundingRect() will be used to find out their bounding boxes. Figure 2 shows the radial basis method structure.

**Figure 3:**
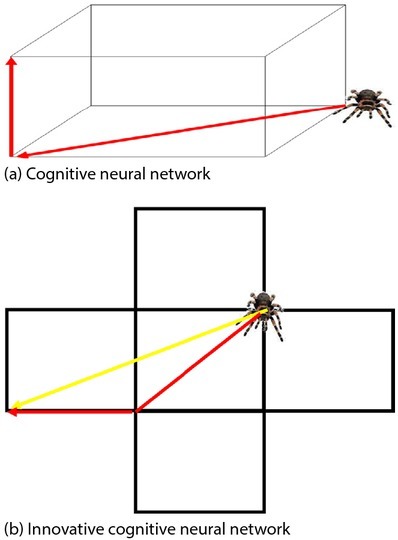
Cognitive neural network and innovative Cognitive neural network comparison

Cognitive neural network network and BP network comparison:

The output of cognitive neural network is a linear sum of weighted unit, and the learning speed has accelerated. The innovative RBF network uses the sigmoid() function as an activation function so that the neuron has a large input visible area. Figure 3 Cognitive neural network and innovative cognitive neural network comparison. The input area of neurons is very small and therefore more radial basis neurons are needed.

## 4 Application and Algorithm simulation test

### 4.1 Architecture selection

The digital pictures used for training and testing are all grayscale images with a resolution of 20*36, as shown in table 1. After each image is binarized, it is divided into 16 blocks of equal size, and the number of one’s is counted, and is taken as the feature vector of each picture.

**Table 1:** Basic situation of the subjects

x1	x2	R1(X)	R2(X)
1	0	0.3679	0.3679
0	1	0.3679	0.3679
0	0	0.1353	1
1	1	1	0.1353

Step 1: Start with the number of the hidden neurons by 1.

Step 2: Perform k-means clustering to find the location of centers for the hidden numbers.

Step 3: Select the width of each neuron to be half of the maximum distance between the center itself and other neurons.

Step 4: Using pseudo-inverse method to obtain the weight.

Step 5: For a selected Q value, compute the current methods error bound by the following equation.

(7) $$R_{\text{emp}}+ST-SM=\frac{1}{N}\sum_{i=1}^{N}{\left(f_{\theta }\left(z_{i}\right)-F\left(z_{i}\right)\right)}^{2}+E_{s_{Q}}\left(\Delta y^{2}\right)$$

Step 6: Find the minimum error bound, and output the corresponding hidden neuron’s number.

### 4.2 Method Construction and Training

**Figure 4:**
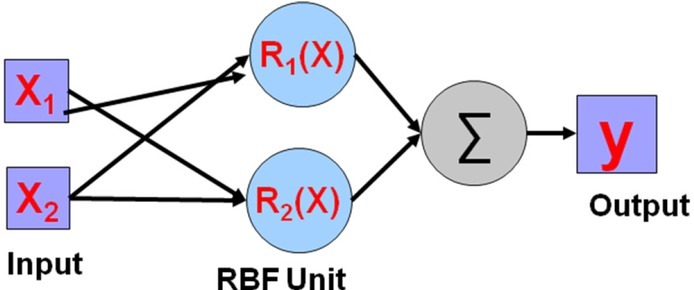
The innovative cognitive neural network method in digital image recognition

The method used for digital image recognition in this paper has one input layer, each output layer has 16 nodes, the number of nodes is 1. Among them, the excitation of the first output layer is sigmoid, and the excitation of the second one is an S-type tangent function [8]. The global error is set to 01001. 300 training samples are input into the cognitive neural network method for training. The innovative cognitive neural network method in digital image recognition are shown in the follow (see Figure 4).

**Figure 5:**
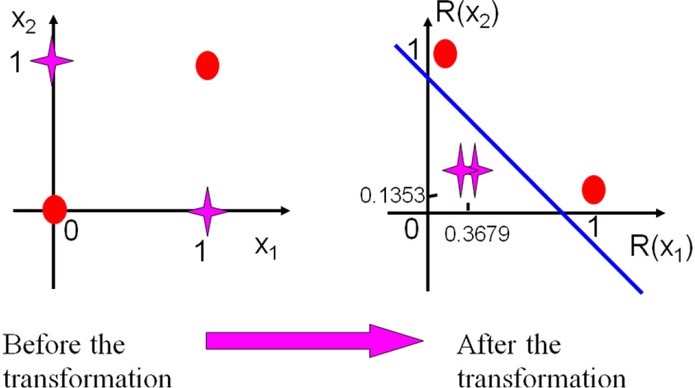
Changes in the transformation of innovative cognitive neural network x1 represents an independent variable and R(x) represents a dependent variable.

### 4.3 Method performance test

The 100 test samples have input into the trained method, and the comparison between the recorded output results and the target results showed that the correct rate was 92% [9]. The innovative cognitive neural network method has a high recognition rate in image recognition.

**Figure 6:**
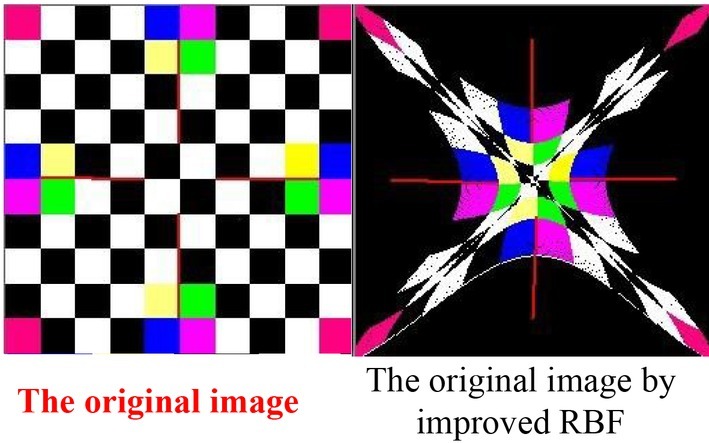
Changes in cognitive neural network during the recovery period

The training results are shown in Figure 6. For the given experimental lattice image, all the dots in the last line are white pixels, so that after the transformation, a white stripe is added at the bottom of the pattern than the result of the reference experiment report [10]. In Fig. 6, the innovative cognitive neural network learning algorithm has reached the preset precision after 1047 iterations. It is the innovative cognitive neural network learning algorithm, that is, the convergence performance is obviously better than the original cognitive neural network learning algorithm. The next step is to extract the seven invariant moments of the image, and then use the improved neural network (BP) to study the image target recognition algorithm. The results show that the algorithm has better recognition effect and faster speed, which can be applied to small images.

### 4.4 Discussions

The potential application prospects in the areas of office automation, automatic meter identification, and bank check recognition are very broad. The problem of Arabic numerals recognition can be attributed to a supervised learning problem, that is, hoping to learn the knowledge or information needed for classification from a given sample feature set, thereby constructing a decision surface. This paper presents an innovative cognitive neural network method application in digital image recognition. Each point of the graph has transformed, and the original color of the transformed new coordinates has assigned to the original point. As for why no point has converted to this coordinate, it is because the point coordinates are all integers and are discrete points. After the transformation, they are real numbers and need to be integers. Inevitably, they need to be rounded. In this operation, some points may not have other points to change to that point, thus forming voids or stripes. The IDW method can effectively separate the source image signal from the mixed signal and maintain the waveform of the source signal with high accuracy, thus laying the foundation for the next step of recognition.

## 5 Conclusions

With the continuous development of social informatization, the issue of automatic recognition of Arabic numerals has gradually attracted the attention of researchers in related fields. This paper uses the innovative cognitive neural network method algorithm for digital image recognition. First, certain number of training samples have used to train the method to obtain an RBF method that satisfies the global error index. Then, the test sample has used to test the recognition rate of the method. The test result is over 90%. The test results show that the innovative cognitive neural network method has a significant improvement in the convergence speed compared with the original cognitive neural network method, and the innovative cognitive neural network method has a high accuracy in digital image recognition.
